# A Quantitative Assessment of Sustainable Development Based on Relative Resource Carrying Capacity in Jiangsu Province of China

**DOI:** 10.3390/ijerph15122786

**Published:** 2018-12-09

**Authors:** Jinbi Yang, Hao Ding

**Affiliations:** School of Business, Jiangnan University, 1800 Luhu Avenue, Wuxi 214122, China; 609844124@163.com

**Keywords:** relative resource carrying capacity, model improvement, sustainable development

## Abstract

Unbalanced development is an urgent issue that needs to be resolved in the sustainable development strategy of Jiangsu Province, which inhibits Jiangsu’s industrial transformation and upgrading. A relative resource carrying capacity model is extended based on resource carrying capacity to analyze the resource carrying capacity of the different regions of Jiangsu Province. Three indicators of water resources, land resources, and energy resources are included in the natural resources. In social resources, factors of population quality are included in the analysis scope. Based on the improved model, this paper analyzes the relative resource carrying capacity of Jiangsu Province. The results show that: (1) under both traditional resource carrying capacity model and the improved model, Jiangsu Province has a surplus population; however, there is a certain difference between the result from two modules; (2) contributions of environmental resources, economic resources, and social resources to the comprehensive carrying capacity of resources is obviously higher than the contributions of natural resources; and (3) significant regional differences exist in relative resource carrying capacity within Jiangsu Province between the southern region and the middle region, in which the capacity is surplus to the population demand, and the northern region, in which the capacity is overloaded.

## 1. Introduction

Manufacturing is an essential part of the national economy. China has been devoted: (1) to achieving sustained and rapid development of the manufacturing industry; (2) to promoting industrialization and modernization processes; (3) to enhancing the comprehensive national strength; and (4) to supporting its aspiration to be the world’s leading power [1]. However, compared to other developed economies, such as Germany and Japan, there is still a gap in China’s manufacturing industry; thus, the task of transforming China’s manufacturing industry is urgent and arduous. Resource consumption and pollution of the environment for the benefit of manufacturing threaten the stability and sustainability of the ecosystem. With the spread of the concept of sustainable development, individuals from all walks of life gradually pay more attention to the protection of resources and environment, and start to understand the importance of coordinated development between resources, environment, economy and society [2]. In terms of sustainable development, the economic system, environmental system, and social system are indispensable subsystems. Therefore, the regional sustainable development is the result of a coordinated development of various systems [3]. In recent years, many scholars in the research field of regional sustainable development adopted the resources carrying capacity model to explore regional coordinated development [4].

“Made in China 2025” is an important strategic plan to improve the development quality and level of the Chinese manufacturing industry in an all-round way in the new international and domestic environment [5]. The Chinese government generates this plan with the expectation of international industrial transformation in the manufacturing industry. According to the plan, China will become a manufacturing power by 2045. As one of the measures of “Made in China 2025”, the government approved five cities in southern Jiangsu (Zhenjiang, Nanjing, Changzhou, Wuxi and Suzhou) as pilot cities. Jiangsu Province is located in the rich Yangtze River Delta; Nanjing, Suzhou, Wuxi, Xuzhou and other 13 cities are under its jurisdiction. Jiangsu is a typical economically developed region, and the economy of Jiangsu Province plays an important role in China [6]. The study on resource carrying capacity and sustainable development of Jiangsu Province can provide a reference for other regions in China.

Due to the large population and limited total resources in China, the population that can be carried by the traditional calculation method is almost always lower than the actual population [7]. Hence, the traditional method of resource carrying capacity is not enough for China whose regional distribution of resources is uneven. Therefore, we should consider the relative resources carrying capacity, which focuses on how to make a reasonable allocation on limited resources. In order to seek the path of Jiangsu’s industrial transformation and upgrading, and promote the coordinated development of population, resources and environment in Jiangsu, the relative resource carrying capacity model is expanded based on the existing resource carrying capacity framework in our paper. Three indicators of water resources, forest resources and energy resources are included in the natural resources subsystem, while population quality factor is included in the analysis category in the social resource subsystem. Relative resource carrying capacity status of Jiangsu Province and 13 cities is analized based on improved model, and reveals the interrelation and evolution law of population, resources and environment in Jiangsu Province. It will provide not only a reference for realizing the sustainable development of Jiangsu Province, but also theoretical and practical significance for expanding the background of the research on relative resources carrying capacity and exploring the sustainable development of other human-land relations.

## 2. Literature Review

Carrying capacity is defined as the function of characteristics of both the area and the organism [8]. Resources carrying capacity considers the relationship between resources, environment and ecology from the perspective of human survival and development [9]. It indicates that the population supporting capacity of basic survival and development relies on the resources quantity and quality in the particular country or region [10]. Park and Burgess [11] formally put forward the concept of carrying capacity in the study of human ecology, that is, “under a specific environmental condition (mainly refers to the living space, the combination of nutrients, sunlight and other ecological factors), the highest limit of the number of individuals existence.” Huang [12] first proposed the concept of land carrying capacity. Then Hu [13] linked the concept of land carrying capacity with the theoretical maximum constant of the logistic curve. Millnigtno and Gifford [14] used the multi-objective decision analysis method to measure the capacity of land resources in Australia. Wang [15] and Zeng [16] focused on water environmental carrying capacity and aquatic ecological carrying capacity. Bishop [17] and Holling [18] conducted relevant researches on environmental carrying capacity and ecological carrying capacity respectively. Slesser [19] proposed the ECCO model (Enhancement of Carrying Capacity Options) as a calculation method for new resource and environmental carrying capacity. From this we can see that the research of carrying capacity has developed in depth from a single dimension carrying capacity to a multi-dimensional carrying capacity.

Prior research on the carrying capacity of resources usually uses a closed and isolated system to examine the carrying capacity of a resource on the population from a single point of view. Instead, the relative resource carrying capacity is based on one or more larger areas, and uses a more open and practical perspective to investigate the area of population carrying capacity of resources. It also expands the research scope of resources carrying [20]. Zhu [21] first put forward the idea of relative carrying capacity of resources, and proposed the modeling framework of “P-E-R” regional matching model of population, economy and resources. Afterwards, the concept of relative resource carrying capacity and model were further constructed [20]. Then, the model was adopted to analize the relative resource carrying capacity of the Yangtze River Basin, Northeast China, Jiangsu, Jilin, and Ningxia respectively [22,23,24,25,26]. Li [27], Huang [7], and Sun [28] have carried out model improvement research and calculation method design aiming at the deficiency of relative resource carrying capacity model. Similarly, Wang [29] constructed a relative resource carrying capacity model and coupled coordination model to conduct a patio-temporal analysis of the coordinated development in the “economy-resource environment-society” system. Generally, resources include natural resources (i.e., resource utilization methods, utilization efficiency, etc.), environmental resources (i.e., environmental protection concept, application of environmental technology and products etc.), economic resources (i.e., production efficiency, consumption patterns, and economic development concepts etc.) and social resources (i.e., social interaction, social civilization level etc.) [30]. With the development of science and technology and the integration of economy and society, manufacturing and life of people in a specific area are less and less dependent on the resources in the region [21], and the natural factors have limited effects on the relationship between human and land in the region. Therefore, the research scope of resource carrying capacity should be extended to better reflect the population carrying capacity of resources in a multi-dimensional resource perspective [26,31,32].

Until now there has been no consensus regarding the research of relative resource carrying capacity; research on the calculation model of relative resource carrying capacity and the evaluation index of resource carrying capacity is still under exploration. There are only a few papers that conduct empirical analysis of relative resource carrying capacity and sustainable development in Jiangsu Province. Therefore, this paper focuses on the expansion of the relative resource carrying capacity model and the empirical analysis of the carrying capacity of Jiangsu Province. Based on the analysis of the relative resource carrying capacity, this paper extends the resource range of the relative resource carrying capacity according to system theory, to analyze the situation of the relative resource carrying capacity of Jiangsu Province from 2001 to 2015, and to analyze the relative resource carrying capacity of 13 cities in Jiangsu Province in 2015 in order to reveal the trend and characteristics of resource carrying capacity in Jiangsu Province.

## 3. Model

### 3.1. Expansion of Relative Resources Carrying Capacity Model

Rich water resources and land resources not only provide a beautiful ecological environment for Jiangsu Province, but also provide favorable conditions for the development of ecological industry, such as the “Taihu Lake Ecological Economic Community” [33]. By 2013, there are 133 kinds of mineral resources have been discovered in Jiangsu Province, of which 67 types have been identified and another 66 still have not been identified. Coal, oil, natural gas, iron and other energy minerals and metal mineral reserves are low in China. Therefore, the abundant water resources, land resources, and energy resources in Jiangsu Province have strong economic effects and offers a large carrying capacity on the population. In view of this, water resources, land resources and energy resources are included in the analysis of natural resources carrying capacity, so as to reflect the carrying capacity of natural resources on the population of Jiangsu Province more comprehensively.

Population quality usually refers to the comprehensive quality and ability of the population of a country or region, including physical quality, scientific and cultural quality and moral quality [34]. It is believed that the natural resource subsystem and the environmental resource subsystem are the main constraints for sustainable development of carrying capacity, while economic resources subsystems and social resources are the basis for the sustainable development of carrying capacity [28]. Although some scholars apply system theory to incorporate the population resource subsystem into the relative resource carrying capacity concept [28], the role of the population resource subsystem in the whole sustainable development system is difficult to clearly clarify. As the main body of social and economic activities, the behavior of population will not only affect population quantity, population structure and population migration in population resources subsystem, but also affect the subsystems of resources, environment, economy and society [29]. For example, the potential of resources can be transformed into the actual carrying capacity only by improving the level of science, technology and productivity through the development of economy [32]. Therefore, the population resource subsystem should be the target carrier and dynamic condition for the sustainable development of carrying capacity. Population quality is not only representative of the overall quality of the population resources subsystem, but also the main way for the population to play a role in the other subsystems, and will eventually affect the comprehensive carrying capacity on resources.

### 3.2. Modeling

According to the relative resource carrying capacity [29], this paper makes appropriate adjustments to the model based on the natural ecological characteristics of the study area. This paper regards water resources, crops planting area, and energy resources as indicators for measuring natural resources. Industrial wastewater discharges are served as indicators for measuring environmental resources. GDP is treated as indicators for measuring economic resources, and the total retail sales of social consumer goods and high school and above education population are regarded as indicators for measuring social resources. We construct an extended “W (water)-L (land)-4E (energy, environment, and economy)-C (sales of social consumer goods)-H (high school and above education population)” relative resource carrying capacity model referring to the research of Zhu [21], Huang [20], and Sun [28]. Although the determination of weights in the weighted summation model of relative resource carrying capacity is subjective, it is not suitable for the geometric form of relative resource carrying capacity model in view of the abundant water resources and cultivated land resources and the lack of energy resources in Jiangsu Province. Therefore, the weighted model of relative resource carrying capacity is still selected:

*Cs = W*_1_*C_rwle_*_1_* + W*_2_*C_re_*_2_* + W*_3_*C_re_*_3_* + W*_4_*C_rch_* (1)

where *Cs* denotes the relative comprehensive resource carrying capacity. *W*_1_, *W*_2_, *W*_3_ and *W*_4_ are the weights of natural resources, environmental resources, economic resources, and social resources in the relative carrying capacity on comprehensive resources.

The relative natural resource carrying capacity is calculated as:

*C_rwle_*_1_*= W*_5_*C_rw_ + W*_6_*C_rl_ + W*_7_*C_re_*_1_ (2)

where *C_rwle_*_1_ denotes the relative natural resource carrying capacity, that is the sum of *C_rw_*, *C_rl_*, and *C_re_*_1_, which includes the water resources, land resources, and energy resources carrying capacity. C_rw_ = I_rw_ × Q_rw_, C_rl_ = I_rl_ × Q_rl_, C_re1_ = I_re1_ × Q_re1_. Q_rw_, Q_rl_ and Q_re1_ are the total amount of water resources, crops planting area, and total energy production in the study area respectively. The calculation formula for the natural resource carrying index is: I_rw_ = P_0_/Q_rw0_; I_rl_ = P_0_/Q_rl0_; I_re1_ = P_0_/Q_re10_. In the formula: I_rw_, I_rl_, I_re1_ are the water resources, land resources, and energy resource carrying index; P_0_ is the population of the reference area; Q_rw0_, Q_rl0_, Q_re10_ are the crop sown area, total water resources and total energy production in the reference area. W_5_, W_6_, and W_7_ are the weights of water resources, land resources, and energy resource resources in the relative natural resources carrying capacity.

C_re2_ means the relative environmental resource carrying capacity. C_re2_ = I_re2_ × Q_re2_. Q_re2_ is the total amount of industrial wastewater discharged into the research area. The calculation formula of environmental resource carrying index is: I_re2_ = P_0_/Q_re20_. In the formula: I_re2_ is the environmental resource carrying index; P_0_ is the population of the reference area; Q_re20_ is the total industrial wastewater discharge of the reference area.

C_re3_ means the relative economic resource carrying capacity. C_re3_ = I_re3_ × Q_re3_. Q_re3_ is the regional GDP of the study area. The calculation formula of economic resource carrying index is: I_re3_ = P_0_/Q_re30_. In the formula: I_re3_ is the index of economic resources; P_0_ is the population of the reference zone; Q_re30_ is the GDP of the reference zone.

The calculation formula of relative social resource carrying capacity is:

*C_rch_ = W*_8_*C_rc_ + W*_9_*C_rh_* (3)

where *C_rch_* denotes the relative social resource carrying capacity, which is the value of the combination of *C_rc_* and *C_rh_*, which includes social consumer goods resources and human capital resources. C_rc_ = I_rc_ × Q_rc_, C_rh_ = I_rh_ × Q_rh_. Q_rc_ and Q_rh_ are the total retail sales of social consumer goods and the number of educated people in high school and above in the study area. The calculation formula for the social resources carrying index is: I_rc_ = P_0_/Q_rc0_; I_rh_ = P_0_/Q_rh0_. In the formula: Irc, Irh are social consumer goods resources and human capital resources carrying index; P_0_ is the population of the reference area; Q_rc0_, Q_rh0_ are the total retail sales of social consumer goods and the number of educated people in high school and above in the reference area. W_8_ and W_9_ are the weights of social consumer goods resources and human capital resources in relative social resources carrying capacity.

According to the specific conditions of the study area, the weights are usually set to reflect the differences of resource matching in different regions and the impact of different resources on the quality of life and development of the population. According to the specific situation of Jiangsu Province and the previous literature [28], the weights are set as: W_1_ = W_3_ = 0.3; W_2_ = W_4_ = 0.2; W_5_ = W_6_ = 0.4, W_7_ = 0.2; W_8_ = 0.6, W_9_ = 0.4.

The type of carrying state: The carrying state can be divided into three states: overload, surplus, and critical, that is P − C_s_ > 0, P − C_s_ < 0, P − C_s_ = 0. Among them, P is the actual population. The formula for the overload rate (or surplus rate) is: R = P′/P. R is the relative resource overload rate (or surplus rate), and P′ is the actual overload population: P′ = P − C_s_.

## 4. Data and Results

**Figure 1:**
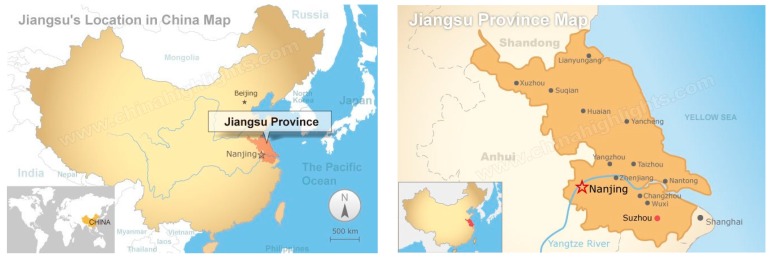
Jiangsu Map.

Jiangsu Province, covering an area of 102,600 km^2^, is located along the eastern coast of China, which has a 954-km-long coastline and a water surface area of 17,300 km^2^ [35]. It is an important economic zone in the Yangtze River delta with 13 prefectural cities, as shown in Figure 1. Gross Domestic Product (GDP) of Jiangsu Province took the second place of China 28 provinces in 2017 [36]. According to differences in economic development, Jiangsu Province is divided into South Jiangsu, Central Jiangsu, and North Jiangsu [37]. The data of population, total water resources, planting area of crops, total energy production, discharge of industrial waste water and total retail sales of consumer goods are derived from the “Statistical Yearbook of China” and “Statistical Yearbook of Jiangsu” from 2001 to 2015 [38].

### 4.1. Comparison between Models before and after Improvement

In the traditional relative resource carrying capacity model, crop planting area, regional GDP, total retail sales of consumer goods in the region, and industrial wastewater discharge are regarded as relative natural resource carrying capacity, relative economic resource carrying capacity, relative social resource carrying capacity, and relative environmental resource carrying capacity. In the improved relative resource carrying capacity model, water resources and energy resources are included into the relative natural resources carrying capacity, and human capital resources are introduced into the relative social resource carrying capacity. The model analysis results before and after improvement (Table 1) show that: based on the analysis of the traditional relative resource carrying capacity model, the comprehensive carrying capacity of relative resources in Jiangsu Province has always been surplus, with an average surplus population of 32 million people in the period 2001–2015; and the relative comprehensive carrying capacity of Jiangsu Province based on the improved model is also in a state of surplus, but the average surplus population is only 22 million people. From a dynamic point of view, the relative resources surplus rates based on the traditional model analysis and the improved model analysis have the same trend, they have always been negative and their absolute values are increasing, which indicates that the surplus scale of resources carrying capacity in Jiangsu Province is increasing. In addition, the existence of certain differences in the two model analyses indicates that the contribution rate of energy resources in natural resources is low, and the contribution rate of human capital resources in social resources is low. Therefore, we should fully consider the advantages and disadvantages of the resources when analyzing the regional resource carrying capacity, otherwise it will enlarge the population carrying capacity significantly, which will bring negative impact to the understanding of the sustainable development in Jiangsu Province.

### 4.2. The Analysis Based on the Improved Model

#### 4.2.1. Relative Comprehensive Resource Carrying Capacity

As far as the whole country is concerned, relative comprehensive resource carrying capacity of Jiangsu Province is in a surplus status from 2001 to 2015, but the size of the surplus population has a downward trend (Table 2). Generally, relative comprehensive resource carrying capacity of Jiangsu Province remains in a surplus status. The average comprehensive resource carrying capacity is 99 million people, and the surplus rate and surplus scale have been relatively stable in recent years; however, the surplus scale decreased from 26 million people at the highest level in 2005 to 23 million people in 2015, and the actual permanent population increased by 4 million people during the period; this shows that with the development of economic and social in Jiangsu Province and the increase of the actual population, the comprehensive carrying capacity of relative resources in Jiangsu Province tends to be stable, and the influence of human activities such as production and living on resources and environment got smaller and smaller from 2001 to 2015.

#### 4.2.2. The Development Trend of Each Resource Carrying Capacity

**Figure 2:**
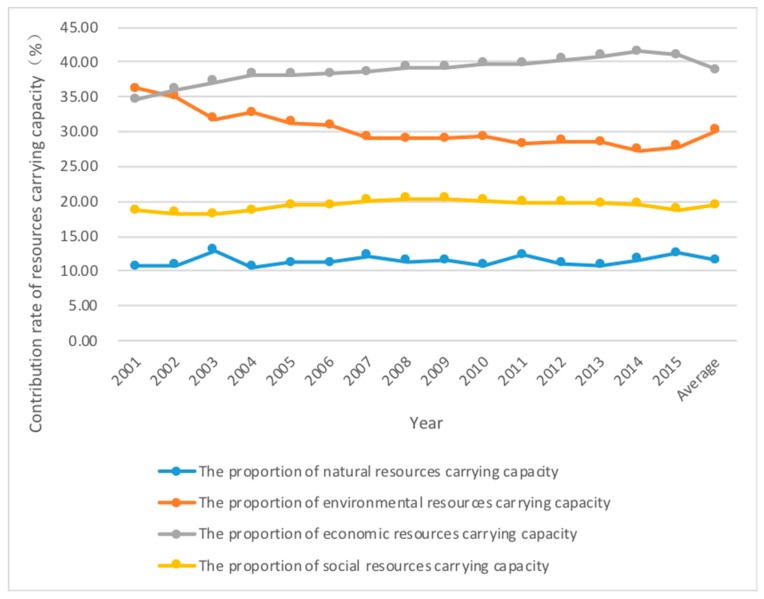
Evolution of relative resources carrying capacity of Jiangsu Province.

The carrying capacity of economic resources contributed the most to the population carrying capacity of Jiangsu Province. Only the carrying capacity of natural resources is overloaded and the contribution to population capacity is the lowest. The environmental resources carrying capacity, economic resources carrying capacity and social resources carrying capacity of Jiangsu province are higher than the actual population, among which the economic resources carrying capacity is the highest. The natural resources carrying capacity is the lowest, lower than the actual population. In the period 2001–2015, the average contribution rate of natural resources carrying capacity in Jiangsu Province is 11.5%, the average contribution rate of relative environmental resources decreases to 30.2%, the average contribution rate of economic resources increases to 38.9%, and the average contribution rate of social resources increases to 19.4% (Figure 2). By the end of 2015, the carrying capacity of the environmental resources was 142 million people, the economic resources carrying capacity was 140 million people, the social resources carrying capacity was 96 million people, and the natural resources carrying capacity was 43 million people. Among the four indicators of resources carrying capacity, only the carrying capacity of natural resources is always lower than the actual population, and the other three items are all higher than the actual population. This shows that environmental resources, economic resources and social resources are the main carriers of the social population for the region, and the relative carrying capacity of natural resources to the population is also rising with the protection of natural ecology.

#### 4.2.3. The Trend of Natural Resources Development

The carrying capacity of natural resources is always in overloading status, but the trend is steady. As we can see: land resources carrying capacity > water resources carrying capacity > energy resources carrying capacity. Land resources carrying capacity is relatively rich, slightly lower than the actual population, which indicates that land resources make a great contribution to the sustainable development of Jiangsu Province. However, the water resources carrying capacity and energy carrying capacity are obviously insufficient, and they are always in deficit status, which indicates that water resources and energy resources are the main factors which restrict the sustainable development of Jiangsu Province.

#### 4.2.4. The Trend of Social Resources Development

The social resources carrying capacity of Jiangsu Province is always in a state of surplus, and presents a steady trend. Social consumer goods resources carrying capacity > human capital resources carrying capacity. The carrying capacity of social consumer goods resources is increasing continuously and is always higher than that of real population, which indicates that social consumer goods resources make a great contribution to the sustainable development of Jiangsu Province. The carrying capacity of human capital resources shows a declining trend, and it started to fall below the actual population after 2012, which indicated that the human capital resources in Jiangsu Province are losing.

### 4.3. Regional Differences of Relative Resource Carrying Capacity among Cities in Jiangsu Province

The process of regional economic development has always been closely related to the local natural conditions and natural resources. Using the country as a reference area, and 2015 as a reference year, the relative resources carrying capacity of a total of 13 cities in Jiangsu Province in 2015 is calculated (Table 3). Table 3 shows that the relative resource carrying capacity of Jiangsu province has an obvious regional disparity.

#### 4.3.1. The Comprehensive Carrying Capacity Is Extremely Abundant (South Jiangsu)

There are five cities, which are Nanjing, Wuxi, Changzhou, Suzhou and Nantong, located in the south of Jiangsu Province. This area is the region that has the highest level of economic development in Jiangsu Province. Rapid economic development has brought a comprehensive carrying capacity that far greater than the population, accordingly, these cities all have extreme surplus capacities. Among them, Suzhou has a largest population of 10 million, and Nantong has a smallest surplus population of 2 million. Suzhou, with the largest surplus in this area, accounts for 93.9% of the total population of the city, and Nantong City has the smallest surplus, whose surplus population accounts for 26.5% of the total population of the city. In addition, we can see that environmental resources carrying capacity reached 42 million people, and the economic resources carrying capacity reached 29 million people in Suzhou. Its comprehensive resource carrying capacity is much higher than the other four cities and is in a status of extreme surplus. It is mainly because of the deep promotion of pollution reduction, the acceleration of transformation and upgrading, the promotion of ecological environment protection and the construction of ecological in Suzhou. The contribution rates of environmental resources carrying capacity, economic resources carrying capacity and social resources carrying capacity to comprehensive carrying capacity of the cities in the region are far higher than the contribution rate of relative natural resources carrying capacity, and the carrying capacity of natural resources is in an overloaded status. This precisely shows that the natural resources of the region is a short board for the carrying capacity of the population, and the government still needs to provide policy guidance. While developing the economy, it is necessary to take into account the protection of water resources and land resources, to improve the efficiency of the utilization of energy resources, to reduce the dependence on natural resources and to improve the carrying capacity of natural resources.

#### 4.3.2. The Comprehensive Carrying Capacity Is Lightly Surplus (Central Jiangsu)

The cities of Huai’an, Yancheng, Yangzhou, Zhenjiang, and Taizhou are located in the central part of Jiangsu Province. The comprehensive carrying capacity of this area is slightly rich. Among them, Zhenjiang has the largest surplus population, which is 1.5 million. The city which has the largest proportion of surplus population is also Zhenjiang, of which the surplus population accounts for 47% of the total population. Huaian city has the smallest surplus population, which is 0.2 million, and accounts for 3.5% of its total population. In this case, the environmental resources carrying capacity of Yancheng reached 11 million, which is much higher than the other four cities. It has obvious advantages in environmental resources. It is necessary to strengthen environmental protection so as to maintain this advantage. However, the comprehensive resource carrying capacity of Huai’an is only 5.04 million people, and the environmental resources and social resources are at a disadvantage. It is necessary to give full play to the advantages of natural resources and economic resources by vigorously developing the economy and improving resource utilization, by reducing the damage to the environment, and also by vigorously introducing talent. The carrying capacity of natural resources and social resources of the region are equal to the actual population, while the carrying capacity of environmental resources and the carrying capacity of economic resources are all in a surplus status. While intensifying environmental protection and vigorously developing the economy in this area, it is necessary to reduce damage to the environment by protecting natural resources and improving utilization. It is also necessary to introduce talents and strengthen social resources.

#### 4.3.3. The Comprehensive Carrying Capacity Is Overloaded (North Jiangsu)

There are 3 cities, which are Xuzhou, Lianyungang and Suqian, located in the northern part of Jiangsu Province. The comprehensive carrying capacity of this area is overloaded. Suqian city, which has the largest population in this area, has an overloaded population of 0.97 million, which represents 19.9% of the total population, which is also the largest rate in this area. The city which has the smallest overloaded population is Xuzhou City, with an overloaded population is 0.05 millon, which represents 0.6% of the total population. The economic development in the region is lagging behind, and the comprehensive strength is not strong. The carrying capacity of resources and social resources in Xuzhou city are in a surplus status; the carrying capacity of environmental resources in Lianyungang city is in a surplus status; the carrying capacity of remaining resources are in an overloaded status. We can see that the overloading in Suqian City is the most serious and the carrying capacity of each resource is all lower than the actual population of the city. The government needs to guide the region and attract investment, to improve economic strength, protect natural resources and the environment, and also needs to attract talent so as to benefit the development of the region.

## 5. Discussion and Suggestions

### 5.1. Discussion

The comprehensive resources carrying capacity of Jiangsu province has been in surplus status during the period from 2001 to 2015, and the surplus degree is rising steadily. As one of the most developed provinces and regions in China, the relative carrying capacity of environmental resources, economic resources, and social resources of Jiangsu Province show a sharp upward trend, and they are always in a surplus status. The carrying capacity of natural resources also shows a gradual increase trend, but it is always overloaded. Moreover, in recent years, the contribution of land resources carrying capacity to the total carrying capacity of natural resources gradually declined. The carrying capacity of water resources and energy resources is less than the carrying capacity of land resources, but their contribution to the resource capacity is slowly increasing. To a certain extent, it reflects that the growth of the relative natural resources carrying capacity of Jiangsu Province is based on the reduction of relative land resource carrying capacity. In addition, the contribution rate of social consumer goods resource carrying capacity to social resource carrying capacity has gradually increased.

Moreover, the human resources carrying capacity is less than the carrying capacity of social consumer goods resources, and its contribution to the social resources carrying capacity has slowly declined. This indicates that the serious loss of human resources leads to a stable contribution of social resources carrying capacity to the relative total resource carrying capacity, and hinders the contribution of social resources to population carrying capacity. In addition, the regional differences in Jiangsu’s comprehensive carrying capacity are also more obvious: the southern part of Jiangsu is in an extreme surplus state; the Suzhou area is in a state of light surplus; and the northern part of Jiangsu is in an overloaded state. This unbalanced development is also a problem that Jiangsu Province needs to focus on in its sustainable development strategy.

### 5.2. Implications and Suggestions

This research analyzed the carrying capacity of environmental resources, economic resources, social resources, and natural resources of Jiangsu Province, where we could learn: (1) the resource bottleneck of industrial transformation and upgrading in Jiangsu Province performs differently in different dimensions; and (2) there are 13 cities in Jiangsu Province, and each city has different industrial structure and resource carrying capacity, which provides decision-making basis for cities to find differentiated transformation and upgrading paths.

Accordingly, the following suggestions are put forward: (1) a new model of resource saving and efficient utilization must be established, adhering to the principle of saving resources, to promote the conservation of natural resources and to improve the utilization efficiency of resources. In particular, strengthening the awareness of land resources protection and macro-protection measures is of great significance to improve the relative natural resources carrying capacity; (2) local government should enhance ecological environment protection and effective investment, accelerate the formation of sustainable development of the ecological security system, and truly make the ecological environment become endogenous to benefit the people, attract the elements, and centralize industry; (3) enterprises should speed up the adjustment of industrial structure, change the mode of economic growth, and rely on scientific and technological progress to improve resource utilization and promote economic development; (4) local government should strengthen infrastructure and urbanization, increase social resources such as education, culture, medical care, and pensions to meet the needs of the society, and improve the social service system and social consumption levels; and (5) from the perspective of regional disparity, the economic level of southern Jiangsu is generally higher than that in the north, so its relative resources carrying capacity is significantly higher than that of northern region. Therefore, speeding up the economic development in northern and narrowing the economic gap between the northern and southern areas has become an important part of the sustainable development of Jiangsu province in the future.

## 6. Conclusions

Against the background of China’s seeking the path of Jiangsu’s industrial transformation and upgrading, this paper aims to analize the resource carrying capacity of Jiangsu Province to promote the coordinated development of population, resources and environment in Jiangsu. The relative resource carrying capacity model is expanded based on the existing resource carrying capacity framework. The results indicate that the resources carrying capacity of four dimensions (i.e., environmental resources, economic resources, social resources, and natural resources) of Jiangsu province are in the surplus status, but perform differently.

## Figures and Tables

**Table 1:** Comparisons of comprehensive resource carrying capacity of Jiangsu Province from 2001 to 2015.

Year	P	The Traditional Model				The Improved Model			
		C_s_	P′	R	The Type of Carrying State	C_s_	P′	R	The Type of Carrying State
2001	73.59	102.94	−29.35	−39.89	surplus	94.54	−20.95	−28.47	surplus
2002	74.06	102.75	−28.70	−38.75	surplus	93.29	−19.23	−25.97	surplus
2003	74.58	102.33	−27.76	−37.22	surplus	94.61	−20.03	−26.86	surplus
2004	75.23	104.78	−29.56	−39.29	surplus	94.81	−19.58	−26.03	surplus
2005	75.88	112.04	−36.16	−47.65	surplus	102.17	−26.29	−34.64	surplus
2006	76.56	111.92	−35.36	−46.19	surplus	101.86	−25.30	−33.05	surplus
2007	77.23	108.65	−31.42	−40.68	surplus	98.94	−21.71	−28.11	surplus
2008	77.62	109.27	−31.64	−40.76	surplus	98.44	−20.82	−26.82	surplus
2009	78.10	110.79	−32.69	−41.85	surplus	100.75	−22.65	−29.00	surplus
2010	78.69	112.30	−33.61	−42.71	surplus	101.57	−22.88	−29.08	surplus
2011	78.99	111.52	−32.53	−41.18	surplus	102.10	−23.11	−29.26	surplus
2012	79.20	111.76	−32.56	−41.11	surplus	100.71	−21.51	−27.16	surplus
2013	79.39	112.03	−32.64	−41.11	surplus	100.34	−20.95	−26.38	surplus
2014	79.60	111.42	−31.82	−39.97	surplus	100.05	−20.45	−25.69	surplus
2015	79.76	113.25	−33.49	−41.98	surplus	102.47	−22.71	−28.47	surplus
Average	77.23	109.18	−31.95	−41.36	surplus	99.11	−21.88	−28.33	surplus

**Table 2:** Relative resources carrying capacity of Jiangsu Province from 2001 to 2015.

Year	C_rwle1_	C_re2_	C_re3_	C_rch_	C_s_	P	P′	R
2001	33.51	170.71	108.87	88.41	94.54	73.59	−20.95	−28.47
2002	33.84	162.88	111.94	84.88	93.29	74.06	−19.23	−25.97
2003	40.86	150.70	117.01	85.56	94.61	74.58	−20.03	−26.86
2004	33.52	154.91	120.51	88.10	94.81	75.23	−19.58	−26.03
2005	38.12	159.37	129.83	99.55	102.17	75.88	−26.29	−34.64
2006	38.28	157.16	130.24	99.35	101.86	76.56	−25.30	−33.05
2007	40.22	143.97	127.22	99.56	98.94	77.23	−21.71	−28.11
2008	37.15	142.89	128.77	100.44	98.44	77.62	−20.82	−26.82
2009	38.68	145.79	131.73	102.36	100.75	78.10	−22.65	−29.00
2010	36.72	148.93	134.49	102.12	101.57	78.69	−22.88	−29.08
2011	41.78	143.74	135.23	101.23	102.10	78.99	−23.11	−29.26
2012	37.27	144.27	135.46	100.19	100.71	79.20	−21.51	−27.16
2013	36.39	143.02	136.60	99.21	100.34	79.39	−20.95	−26.38
2014	38.96	136.48	138.25	97.95	100.05	79.60	−20.45	−25.69
2015	42.64	142.24	139.88	96.34	102.47	79.76	−22.71	−28.47
Average	37.86	149.80	128.40	96.35	99.11	77.23	−21.88	−28.33

**Table 3:** Relative resources carrying capacity of 13 cities in Jiangsu Province in 2015.

Region	P	C_rwle1_	C_re2_	C_re3_	C_rch_	C_s_	P′	The Type of Carrying State	R
Nanjing	8.24	2.48	15.99	19.39	14.46	12.65	−4.41	surplus	−53.60
Wuxi	6.51	1.69	15.15	16.99	9.36	10.51	−4.00	surplus	−61.38
Xuzhou	8.67	6.42	7.55	10.61	9.98	8.62	0.05	overload	0.62
Changzhou	4.70	1.77	8.94	10.52	6.73	6.82	−2.12	surplus	−45.06
Suzhou	10.62	2.53	41.69	28.93	14.04	20.59	−9.97	surplus	−93.91
Nantong	7.30	5.38	10.85	12.27	8.83	9.23	−1.93	surplus	−26.45
Lianyungang	4.47	3.53	5.06	4.31	4.28	4.22	0.25	overload	5.66
Huai’an	4.87	5.03	4.81	5.48	4.63	5.04	−0.17	surplus	−3.45
Yancheng	7.23	8.83	11.16	8.40	6.57	8.72	−1.49	surplus	−20.59
Yangzhou	4.48	3.26	6.28	8.01	5.09	5.66	−1.17	surplus	−26.19
Zhenjiang	3.18	1.82	6.24	6.99	3.91	4.67	−1.49	surplus	−47.06
Taizhou	4.64	3.61	5.48	7.36	4.40	5.27	−0.63	surplus	−13.47
Suqian	4.85	3.98	3.03	4.24	4.08	3.89	0.97	overload	19.93
